# Comprehensive Echographic Evaluation of Insulinoma, From Bedside to Endoscopic Ultrasonography: A Case Report and Brief Review of the Literature

**DOI:** 10.7759/cureus.57793

**Published:** 2024-04-07

**Authors:** Andrea Da Porto, Martina Antonello, Daniele Macor, Roberta Assaloni, Leonardo A Sechi

**Affiliations:** 1 Department of Medical Area, University of Udine, Udine, ITA; 2 Department of Internal Medicine, University of Udine, Udine, ITA; 3 Department of Gastroenterology, Gastroenterology and Endoscopic Unit, Udine University Hospital, Udine, ITA; 4 Department of Internal Medicine, SOC Diabetologia e Cura del Piede Diabetico, Monfalcone, ITA

**Keywords:** hypoglycemia, endoscopic ultrasonography, pocus, ultrasonography, insulinoma

## Abstract

Insulinoma is a neuroendocrine tumor of the pancreas, and its identification with bedside ultrasonography (US) is extremely rare. With the aim of providing a comprehensive description of the main US characteristics of this rare form of neuroendocrine neoplasm, we are here describing an interesting case of a young woman with insulinoma, identified by using both bedside and endoscopic ultrasounds.

## Introduction

Insulinoma is a rare neuroendocrine tumor of the pancreas. The prevalence of insulinoma is estimated at 1-4 cases per one million person-years [1]. Insulinoma is more common in women and usually presents itself as a solitary and small size nodule (<2 cm in diameter). Less frequently, insulinoma is a manifestation of multiple endocrine neoplasia syndrome type I (MEN-I). Traditionally, the clinical presentation is subtle and a cause of nonspecific symptoms, and insulinoma diagnosis is frequently delayed or misdiagnosed with other disorders. After biochemical evidence of fasting hypoglycemia related to hyperinsulinism, localization of insulinoma is essential. In this context, the use of a bedside ultrasound may help clinicians in improving diagnostic speed, thus leading to the quick diagnosis and prompt management of the disease [2]. Point-of-care ultrasonography (POCUS) is an advanced diagnostic ultrasonography (US) that is performed and interpreted by the attending physician as a bedside test that nowadays has entered into the routine clinical evaluation of most patients [3]. Its relatively fast use has made it a valuable potential diagnostic tool in clinical scenarios where a formal radiological investigation may delay the diagnosis and prompt therapy. In this manuscript, we report an interesting case of pancreatic insulinoma, easily (and fortunately) identified early on with bedside US, and we discuss in detail comprehensive echographic characteristics that we found from bedside to endoscopic contrast-enhanced US, even providing high-quality US images for all readers who may be interested.

## Case presentation

**Figure 1:**
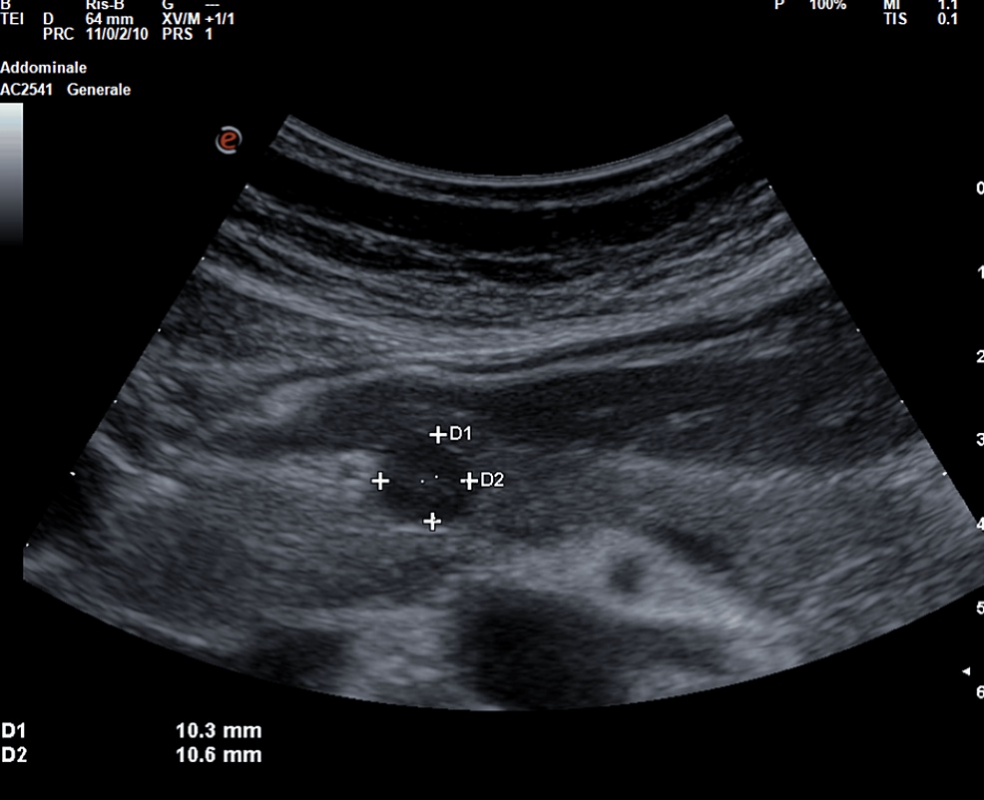
Bedside US from our patient conducted with Esaote MyLab Omega model with abdominal probe and preset showed a well-defined rounded lesion in the isthmic region of the pancreas, hypoechoic compared to the surrounding pancreatic parenchyma, of dimensions 11x10 mm. US: ultrasonography

A 37-year-old woman came to our attention due to her history of episodic and repetitive symptoms, including mental confusion, dizziness, diaphoresis, tremors, palpitations, and occasional loss of consciousness for two years. These symptoms occurred especially during fasting periods and were promptly relieved with eating. Moreover, the patient complained about progressive weight gain (about 10 kg in one year) and a chronic weakness. She had no family history of endocrine disease, and her past medical history was unremarkable. She had no allergies, and she has not been taking any drugs. A physical examination showed a healthy woman with a body mass index (BMI) of 29 kg/m^2^. Main laboratory findings are reported in Table 1, and most of them were unremarkable. Specific tests for the evaluation of glucose metabolism showed a low initial glucose level at 60 mg/dL (74-109 mg/dL), a plasma insulin level at 7.1 μIU/mL (3-25 μIU/mL), and a C-peptide level at 1.49 ng/mL (0.8-7 ng/mL). Bedside US was conducted via the Esaote MyLab Omega model with an abdominal probe and preset that showed a well-defined rounded lesion in the isthmic region of the pancreas, which was hypoechoic compared to the surrounding pancreatic parenchyma, and its dimensions were 11×10 mm (see Figure 1).

**Table 1:** Summary of laboratory test conducted for the diagnostic workup of hypoglycemia. MCV: mean corpuscular volume; AST: aspartate transaminase; ALT: alanine transaminase; Anti-GAD: antibodies against glutamic acid decarboxylase; Anti-IA2: anti-tyrosine phosphatase antibodies; Anti-ZnT8: antibodies against zinc transporter 8; TSH: thyrotropin; FT4: free thyroxine; FT3: free triiodothyronine; FSH: follicle-stimulating hormone; ACTH: adrenocorticotropic hormone

Blood test	Results	Reference
White blood cells	8.08x10^3^/µL	4.00-11.00
Red blood cells	4.72x10^6^/µL	4.20-5.00
Hemoglobin	13.6 g/dL	12.0-16.0
Hematocrit	41.2%	37.0-50.0
MCV	87.3 fL	80.0-94.0
Platelets	396x10^3^/µL	150-400
Creatinine	1.2 mg/dL	0.51-0.95
Sodium	139 mMol/L	136-145
Potassium	2.90 mMol/L	3.5-5.10
Magnesium	0.83 mMol/L	0.66-1.07
Gamma-glutamyl transferase	15 UI/L	6-39
AST	14 UI/L	4-32
ALT	16 UI/L	4-33
Total bilirubin	0.42 mg/dL	0.2-1
Direct bilirubin	0.14 mg/dL	0.00-0.30
Glucose at baseline	60 mg/dL	74-109 mg/dL
C-peptide at baseline	1.49 mg/L	0.90-7.00
Insulin at baseline	7.1 mUI/L	3.0-25.0
Fasting test after three hours		
Glucose	31 mg/dL	74-109 mg/dL
C-peptide	4.39 mg/L	0.90-7.00
Insulin	22.5 mUI/L	3.0-25.0
Autoimmunity		
Anti-GAD	<5.00 UI/mL	0-10
Anti-IA2	<10.00 UI/mL	0-10
Anti-ZnT8	1 UI/mL	0-15
Islet cell antibodies-ICA	<1:4	<1:4
Insulin autoantibodies-IAA	<0.4 UA/ml	<0.4 UA/ml
Pituitary hormones		
TSH	2.38 mUI/L	0.40-4.00
FT4	9.61 pg/mL	8.90-17.60
FT3	3.73 pg/mL	2.30-4.20
FSH	7.1 UI/L	Follicular phase: 2.3-12.6
Prolactin	548.1 mUI/L	Not pregnant: 46.4-642.4
ACTH	15 pg/mL	5-49
Cortisol of the morning	379 nMol/L	150.0-650.0
Urine for sulfonylurea screen	Negative	

**Figure 2:**
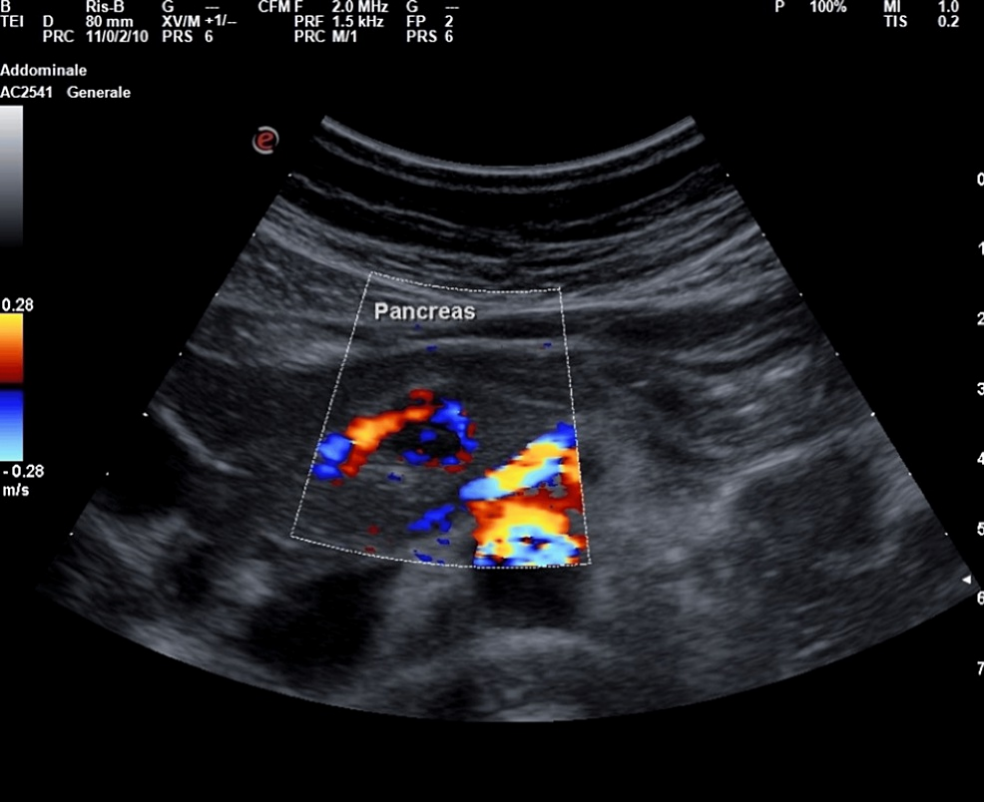
Bedside US of our patient conducted with Esaote MyLab Omega model with abdominal probe and preset; the lesion showed intense peripheral vascularization at the color signal taking the shape of a short rod. US: ultrasonography

At color Doppler evaluation, the lesion showed intense peripheral vascularization at the color signal (see Figure 2), a typical characteristic of large insulinomas and blood flow in the shape of a dot or short rod, as in the cases described in the literature by An et al. [4].

**Figure 3:**
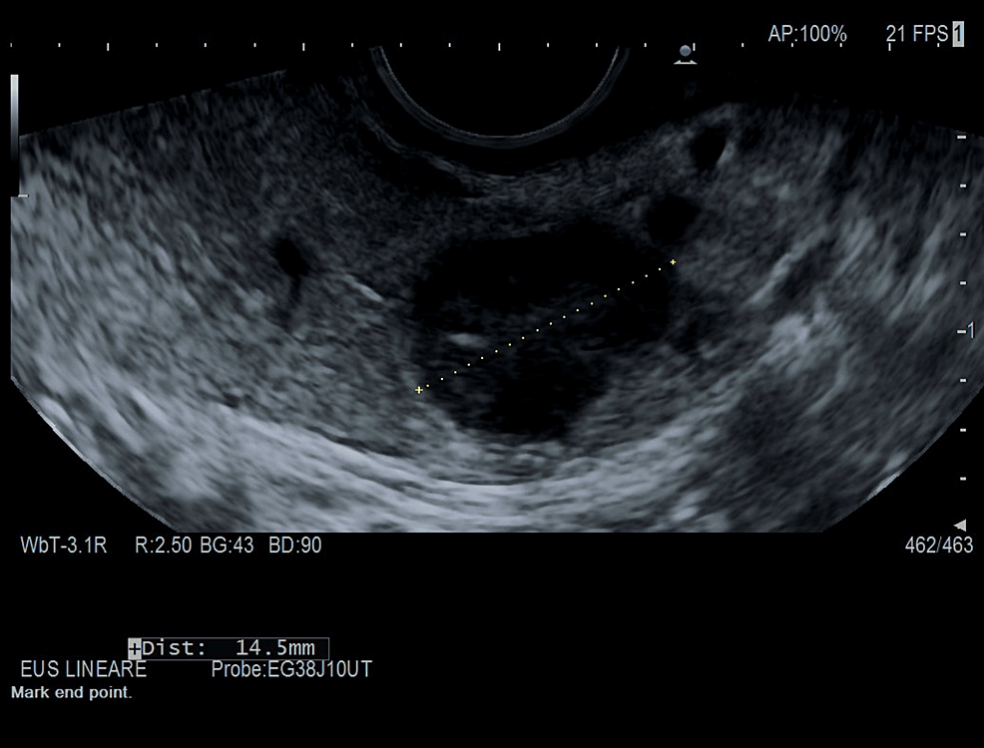
EUS of our patient conducted with EG38-J10UT sector echoendoscope, at the level of the pancreatic isthmus, roundish neoformation, hypoechoic of 13.8 mm × 12.8 of maximum size. EUS: endoscopic ultrasound

**Figure 4:**
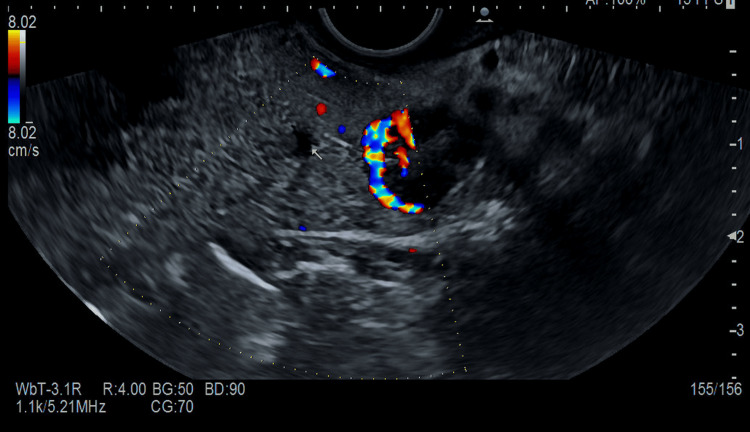
EUS of our patient conducted with EG38-J10UT sector echoendoscope, at the level of the pancreatic isthmus, roundish neoformation, hypervascular to the color Doppler. EUS: endoscopic ultrasound

**Figure 5:**
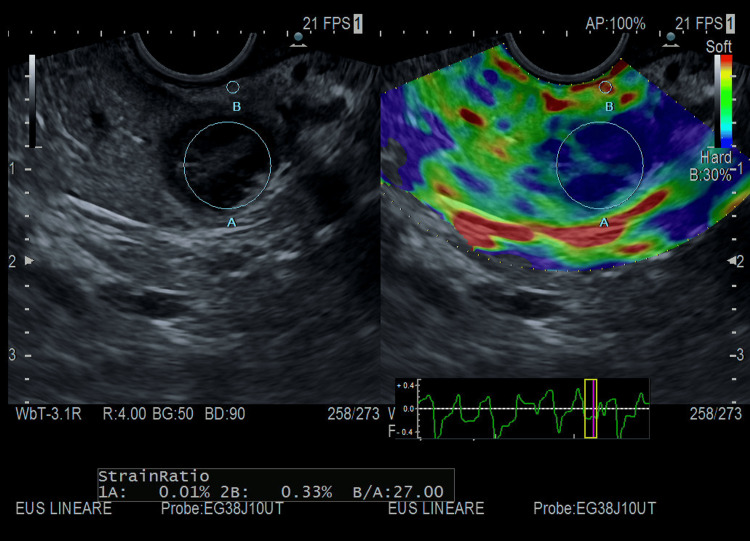
EUS of our patient conducted with EG38-J10UT sector echoendoscope, at the level of the pancreatic isthmus, roundish neoformation, hard to the elastography (SR 27). EUS: endoscopic ultrasound

Thus, we conducted a 48-hour supervised fasting test that produced symptomatic hypoglycemia with hyperinsulinemia at three hours. Urine for the sulfonylurea screen was negative. An abdominal computed tomography scan with contrast demonstrated a well-defined hypervascular lesion involving an uncinate process of the pancreas measuring 15 mm in diameter with enhancement during the arterial phases of contrast bolus, but without liver metastasis or intra-abdominal lymph nodes. A few days later, the patient underwent an endoscopic ultrasound (EUS), which was conducted via the Hitachi EG38-J10UT sector echoendoscope with frequencies between 5 and 10 MHz (see Figure 3, Figure 4, and Figure 5), which confirmed the presence, at the level of the pancreatic isthmus, of roundish neoformation, hypoechoic, hypervascular to the color Doppler, and hard to the elastography (SR 27) of 13.8 mm × 12.8 of maximum size (see Figure 5). After administration of a US contrast agent (SonoVue 1 fL), the lesion presented rapid arterial wash-in and rapid wash-out in the framework referred to as the neuroendocrine tumor. A histologic sample collected with fine needle biopsy during EUS confirmed the diagnosis of a slow-grade neuroendocrine tumor compatible with insulinoma. The patient subsequently underwent radiofrequency ablation surgery with complete remission of hypoglycemic symptoms.

## Discussion

A transabdominal US demonstrated to be a noninvasive, economical, and easily reproducible technique to quickly identify pancreatic neuroendocrine tumors as insulinoma [5]. However, the transabdominal US could not be considered a gold standard imaging due to its strong technical limitations (e.g., a patient's habitus, insulinoma's low dimensions) [4-6] and a poor spatial resolution that seldom makes hard-to-distinguish pancreatic lesions from intestinal loops and peripancreatic lymph nodes [4,5]. Because of these limitations, the sensitivity of the transabdominal US is low, ranging from 9% to 65% depending on the size of the insulinomas [4]. To the best of our knowledge, to date, there are very few reports providing high-quality images and proper descriptions of echographic characteristics of insulinoma revealed by bedside transabdominal US [4,7,8]. A case series described conventional US features in nine patients with histologically proven insulinoma [4]. When detected by conventional US, it typically appears as a rounded hypoechoic formation with well-defined limits [4], with a homogeneous echo structure without calcified areas or signs of central necrosis or cystic degeneration. The lesions were typically single with a low mean diameter of 15.3±2.4 mm (range 7-32 mm). Color Doppler flow imaging (CDFI) could show a dot or short stick-like blood flow. In line with previous findings [4] at bedside US evaluation, we identified a single, well-defined rounded lesion, which was hypoechoic compared to the surrounding pancreatic parenchyma, of the dimensions 11×10 mm (see Figure 1 and Figure 2) and localized in the isthmic region. The lesion showed intense peripheral vascularization at the color signal, a typical characteristic of large insulinomas and blood flow in the shape of a dot or short rod. According to our case, the typical EUS presentation of a neuroendocrine tumor is hypoechoic, well-demarcated, round, hypervascular, and homogeneous with internal echo pattern lesions [9,10]. Conversely, nonsecretory insulinomas can reach dimensions of up to 10 cm, causing a mass effect with dilatation of biliary and pancreatic ducts or echo signs of invasion of surrounding structures [9].

## Conclusions

The identification of insulinoma represents a challenge for the clinician, so with our case report, we want to share with the readers the typical US features of insulinoma. We also want to describe the potential of bedside transabdominal US as a noninvasive, economical, and easy technique for the initial diagnostic workup of recurrent hypoglycemia.
